# Motivations for responses to ostracism

**DOI:** 10.3389/fpsyg.2015.00040

**Published:** 2015-02-03

**Authors:** Eric D. Wesselmann, Dongning Ren, Kipling D. Williams

**Affiliations:** ^1^Department of Psychology, Illinois State UniversityNormal, IL, USA; ^2^Department of Psychological Sciences, Purdue UniversityWest Lafayette, IN, USA

**Keywords:** ostracism, social exclusion, rejection, pro-social behavior, anti-social behavior

Ostracism (being ignored and excluded) and other forms of interpersonal rejection threaten individuals' physical and psychological well-being (Williams and Nida, 2011). Researchers often use the terms ostracism, social exclusion, and rejection interchangeably, but there are theoretical and empirical debates about the differential effects of these phenomena (Smart Richman and Leary, 2009; Williams, 2009; Bernstein and Claypool, 2012). We acknowledge these debates but choose to use the term *ostracism* ubiquitously for simplicity because most of the outcomes we discuss are similar across the phenomena. Most individuals experience these threats at least once during their lives, and some individuals experience them daily (Williams, 2009).

Regardless of the mode or source by which the event occurs, ostracism threatens basic psychological needs (belonging, control, meaningful existence, and self-esteem; Nezlek et al., 2012; Wesselmann et al., in press). Williams (2009) posits a temporal structure to ostracism's effects. In Stage 1, ostracism's basic need threat is ubiquitous with few situational or dispositional moderators (Wesselmann et al., in press). Williams's model is motivation-focused; after the initial threat occurs, individuals should be motivated to recover by fortifying their threatened needs. Stage 2 focuses on cognitive and behavioral processes ostracized individuals use to recover. Stage 3 argues that chronically ostracized individuals withdraw socially and experience extreme psychological and physical damage. We will now focus on relevant Stage 2 research and then discuss suggestions for future research on Stages 2 and 3.

## Stage 2: reflection and recovery

Experimental data suggest that recovery can begin within minutes after ostracism occurs and participants use multiple cognitive and behavioral strategies to recover their thwarted basic needs. Individuals' *cognitive strategies* often focus on attributions for why ostracism occurred and ways to remedy the situation (Williams, 2009). Wirth and Williams (2009) found that attributions influence recovery speed: Individuals attributing ostracism to an experimentally contrived group membership recovered from ostracism quicker than individuals who attributed ostracism to a permanent group membership (i.e., gender; also race in Goodwin et al., 2010; c.f., Masten et al., 2011). Another effective strategy involves encouraging participants to recall ostracism from an outsider's (compared to first-person) perspective (Lau et al., 2009). Also, research demonstrates that priming feelings of physical invulnerability reduces the need for ostracized participants to seek various cognitive and interpersonal recovery options (Huang et al., 2013). Self-construal also facilitates recovery from ostracism: Individuals who have higher interdependent/collectivistic self-construals (i.e., define themselves in terms of social relationships) can recover quicker from ostracism compared to individuals who are lower in these construals (Ren et al., 2013; Pfundmair et al., 2015). Finally, reminding someone of positive social relationships, symbolic/parasocial relationships, or religious/spiritual affiliations facilitates recovery from ostracism (Gardner et al., 2005; Twenge et al., 2007; Epley et al., 2008; Derrick et al., 2009; Aydin et al., 2010, 2012; McConnell et al., 2011; Laurin et al., 2014).

Research on *behavioral strategies* focus mostly on pro- or anti-social behaviors and how they facilitate basic need recovery (Williams, 2009). Experimental research demonstrates that ostracized individuals respond more *pro*-socially than included individuals; they attend more to social information relevant to inclusion (Pickett et al., 2004; Bernstein et al., 2008; Böckler et al., 2014), work harder on group tasks (at least among women participants; Williams and Sommer, 1997), focus more on re-inclusion (Maner et al., 2007; Molden et al., 2009), and show increased sensitivity to social influence (Williams et al., 2000; Carter-Sowell et al., 2008; Riva et al., 2014b). Ostracized individuals also respond more *anti*-socially than included individuals. Ostracized individuals respond aggressively toward another person regardless of whether this person ostracized them. Researchers have measured aggression using diverse methods, such as temptations for physical and social aggression, negative evaluations, unpleasant noise, and ostensibly forcing someone to eat hot sauce (Twenge et al., 2001; Buckley et al., 2004; Warburton et al., 2006).

These two behavioral patterns seem contradictory, but Williams (2009) theorizes that each type of behavior should be linked to the specific psychological needs threatened by ostracism. Pro-social behaviors should be more likely to fortify *inclusionary* needs (belonging and self-esteem) because these behaviors are more likely to achieve re-inclusion; anti-social behaviors should be more likely to fortify *power/provocation* needs (control and meaningful existence) because these behaviors will likely provoke acknowledgement from the ostracizers (see also Gerber and Wheeler, 2009). Ostracized individuals should focus on fortifying whichever need group is most salient to them; ostracized individuals who are unlikely (or unable) to be re-included into a group should focus more on fortifying power/provocation needs (via anti-social behavior) because these needs would be easier to fortify than inclusionary needs (Williams and Wesselmann, 2011).

Experimental research supports Williams's need fortification argument, specifically for control and aggression. Warburton et al. (2006) demonstrated that fortifying ostracized participants' control need immediately after ostracism reduced their aggressive responses to the same level as included participants, whereas unfortified ostracized participants continued to aggress. Schoel et al. (2014) found that ostracized individuals' threatened control (but not the other needs) mediated the ostracism → aggression effect. Other research investigates inclusionary needs and pro-social behavior. Ostracized participants afforded re-connection opportunities behaved less aggressively than ostracized participants not afforded this option (Twenge et al., 2007; DeWall et al., 2010). Pfundmair et al. (2014) investigated the interaction between self-construal and oxytocin (a hormone typically linked to pro-social behavior) on ostracized individuals' reactions (specifically, belonging and self-esteem threat). They found that collectivistic-oriented individuals exposed to oxytocin showed reduced need threat compared to individuals exposed to the placebo, suggesting that simple hormonal cues of affiliation can provide temporary relief from ostracism (at least for individuals who emphasize social relationships in their self-concept). Finally, Bernstein et al. (2010) demonstrated that ostracized participants showed more desire to interact with new sources of affiliation than included participants; this pro-social orientation was mediated by ostracized participants' threatened inclusionary needs. Interestingly, they tested both self-esteem and belonging against each other in a multiple-mediation model and found that self-esteem was a stronger mediator than belonging, suggesting potential nuances within each need cluster that future research should investigate.

### Future research questions

#### Paradoxical responses?

Future research needs to address directly these two conflicting behavioral responses. Often, researchers only give participants one behavioral option. Because of this, it is hard to rule out the possibility that ostracized participants are simply responding more extremely than included participants using whatever option they are given because it is the only option they have to fortify some of their basic needs. Some behavioral measures can be interpreted as pro- or anti-social depending on how participants respond (Gerber and Wheeler, 2009). For example, allocating high amounts of hot sauce to someone who hates spicy food fits the conceptual definition of aggression, but the experimenter instructs participants that they *have* to allocate some amount (whether they choose a small or large amount). Thus, participants who give a small amount could be interpreted as either being *less* aggressive or potentially *more* pro-social because they are obeying the experimenter nominally but also not subjecting the target to unnecessary discomfort. Further, how do researchers categorize participants who actively choose not to allocate any hot sauce at all? Is this simply lack of aggression or also an independent pro-social behavior toward the target?

Recent evidence suggests these two responses might coexist: ostracized individuals seek social connections to an interacting partner (pro-social) and devaluate the same target (anti-social) simultaneously (Sommer and Bernieri, 2014). Gerber and Wheeler (2009)'s meta-analysis found that when forced to choose, participants usually favored anti-social (i.e., control-focused) options. Domachowska et al. (2014) found that ostracized participants preferred higher impact responses (either pro- or anti-social), toward new individuals. They only preferred anti-social responses toward the ostracizers. These findings suggest a complex relationship between behaviors, need satisfaction, and contextual factors. Future research should consider merging these findings with other theoretical models. For example, the Meaning Maintenance Model (Heine et al., 2006) argues that when individuals experience threats to their sense of meaning, they seek recovery either through re-affirming meaning in the threatened domain or indirectly by affirming a symbolically-related domain. Ostracism research typically focuses on threats to four basic needs that are conceptually distinct but inter-correlated (Williams, 2009). It is possible that ostracized individuals can focus on fortifying one specific need (or cluster) and indirectly fortify the others by proxy. Thus, anti-social behaviors may fortify control the most but also may fortify the other three needs in smaller degrees.

Other threat-focused models [e.g., rejection-based threats, Smart Richman and Leary (2009), or threats more broadly, Jonas et al. (2014)] may also offer interesting ways of understanding when and why individuals respond pro- or anti-socially. Jonas et al. (2014) argue that there is a temporal structure to psychological reactions to threat, beginning with anxiety and inhibition or avoidance-based behaviors. Their theorized immediate effects converge with Williams's (2009) argument for Stage 1 reactions to ostracism. Jonas et al. (2014) argues that certain reactions move beyond immediate inhibition-based responses and facilitate approach-oriented behaviors; these behaviors can either address the threat directly or symbolically. The research focused on Williams's (2009) Stage 2 can be re-framed within this theoretical context. Both pro-social and anti-social behaviors could be considered approach-oriented; although pro-social behaviors are the most likely to achieve re-inclusion and primarily fortify the inclusionary needs, anti-social behaviors can also facilitate recovery via the power/provocation needs. The research on cognitive strategies can also be considered approach-oriented in that they either actively help individuals recover need satisfaction through attributional reframing or address the threat symbolically via affirming one's other interpersonal or parasocial relationships.

Researchers could also measure theoretically meaningful individual difference variables to test potential moderation of the anti-social/pro-social paradox. For example, ostracized participants who have a higher dispositional need to belong (Leary et al., 2013) should be more likely to favor pro-social responses (linked to inclusionary needs) than anti-social responses (linked to power/provocation needs). Also, ostracized individuals who are oriented more toward long-term rather than short-term future outcomes respond with higher pro-social behavior than those who favor short-term outcomes instead (Balliet and Ferris, 2013). It is possible that a long-term focus would allow ostracized individuals an edge in overcoming an initial impulse to respond with anger and aggression, thus making inclusionary needs the primary focus. Other research demonstrates that socially anxious individuals recover more slowly (Zadro et al., 2006) and respond less pro-socially when ostracized (Mallott et al., 2009). As social anxious individuals find any social interaction aversive, it is possible these individuals would be less likely to want re-inclusion after ostracism and thus inclusionary needs would be less salient to fortify.

Some individual differences should moderate anti-social responses. Individuals higher in rejection sensitivity (i.e., the tendency to expect and easily perceive rejection; Ayduk et al., 2008) or destiny beliefs (Chen et al., 2012) respond more aggressively than ostracized participants lower in these individual differences. Ostracized individuals high in these latter two variables likely assume that their treatment is consistent with how they will be treated in future interactions; thus the power/provocation need cluster should be most salient to them, facilitating anti-social over pro-social behaviors. Interestingly, ostracized individuals who are high in narcissism also respond more negatively to ostracism than other individuals (Twenge and Campbell, 2003). Narcissistic individuals should expect ubiquitous inclusion because of their inflated self-esteem and may assume the ostracism was anomalous. However, this attribution would not explain their increased aggression using our *future expectations* argument. Wesselmann et al. (2010) offer another possibility: Unexpected ostracism provokes more aggression than expected ostracism, likely because it suggests an inability to read social cues accurately which in turn suggests threats to future social inclusion. Narcissists typically prioritize being admired over being liked (Morf and Rhodewalt, 2001), but they would need to accurately read social cues regardless. Thus, unexpected ostracism would still be more threatening than expected ostracism.

#### Chronic ostracism

Williams (2009) developed Stage 3 by synergizing qualitative interviews of chronically ostracized individuals with other related psychological topics. Chronically ostracized individuals who find their attempts at recovery (Stage 2) continually thwarted should become resigned to their fate and face extreme negative consequences (i.e., feelings of alienation, depression, helplessness, and meaninglessness). Further, chronically ostracized individuals may seek solitude or otherwise disengage from relationships to avoid future ostracism or other unpleasant social interactions (Leitner et al., 2014; Wesselmann et al., 2014). We consider this social withdrawal a *flight* response (compared with *fight* responses, i.e., pro- and anti-social behaviors; Williams, 2007; see also Smart Richman and Leary, 2009; Pfundmair et al., 2015). Additionally, a *freeze* response (Williams, 2007) may encapsulate the cognitive, physical, and affective numbness that sometimes characterizes chronic loneliness and extreme social pain manipulations (e.g., being told one will spend their entire lives alone; DeWall and Baumeister, 2006; Blackhart et al., 2009; Bernstein and Claypool, 2012; Riva et al., 2014a). Few studies have considered these latter two options regarding psychological need threat and recovery, especially within Williams's (2009) temporal model. This area begs for future research and should consider combining Williams's (2009) model with other threat-based models (e.g., Smart Richman and Leary, 2009; Jonas et al., 2014) to derive predictions regarding *flight* and *freeze* responses.

Chronically ostracized individuals may also be susceptible to recruitment by predatory/extreme groups because these groups may offer a last bastion of inclusion (Wesselmann and Williams, 2010; Williams and Wesselmann, 2011). Because ostracized individuals are susceptible to social influence involving mundane behaviors it may also occur for extreme behaviors. For example, Kruglanski et al. (2009; Kruglanski and Orehek, 2011) argue terrorists interpret their actions as a quest for *meaning*/significance (also Jonas et al., 2014). Individuals (or groups) who believe that they have been humiliated, marginalized, or ostracized by larger communities may be attracted to extremist groups endorsing terrorist actions to regain a sense of significance or *control* (Kruglanski, 2003). Other research examining mass violence in schools suggest that that actual or perceived ostracism was a primary motivator for perpetrators (Leary et al., 2003) so it is reasonable to extend this rationale to understanding violence perpetrated by groups of disaffected individuals such as terrorist organizations. These questions offer exciting research opportunities for understanding how ostracism's psychological threats can inspire the worst in individuals and how society can combat these effects.

### Conflict of interest statement

The authors declare that the research was conducted in the absence of any commercial or financial relationships that could be construed as a potential conflict of interest.
